# Epilepsy-Induced Neurodegeneration and Its Therapeutic Interventions

**DOI:** 10.7759/cureus.98549

**Published:** 2025-12-05

**Authors:** Samar Kaddoura, Anjali Banerjee, Latha Ganti

**Affiliations:** 1 Biology, Montverde Academy, Montverde, USA; 2 Biology, University of Georgia, Athens, USA; 3 Emergency Medicine and Neurology, University of Central Florida, Orlando, USA; 4 Medical Science, The Warren Alpert Medical School of Brown University, Providence, USA

**Keywords:** bibliometric analysis, epilepsy, epilepsy treatment, neurodegeneration, review article

## Abstract

Epilepsy is a disease that is recognized not only as a disorder of recurrent seizures but also for its association with neurodegeneration. This bibliometric analysis aims to assess the scientific outputs, trends, and key contributors to epilepsy-induced degeneration and the treatments aimed at intervening in neuronal damage. The Web of Science yielded a total of 662 publications between 1989 and 2025. The analysis identifies many key terms associated with epilepsy and neurodegeneration, including seizures, hippocampus, epileptogenesis, and oxidative stress. With the rise in research on this topic in recent years, there is growing potential for developing therapies that may prevent or reverse neurodegeneration in patients with epilepsy.

## Introduction and background

Neurodegenerative diseases are a group of neurological disorders with a variety of symptoms and causes, affecting specific types of neurons and certain brain regions [1]. These conditions typically have unknown origins and progressively worsen over time [1]. Epilepsy is a disease that affects around 50 million people worldwide and about 1.1% of U.S. adults. Epilepsy is defined as a chronic brain disorder characterized by recurrent EEG paroxysms and clinical seizures, caused by excessive neuronal discharges in the brain, according to multiple studies [2,3]. These factors contribute to behavioral changes like anxiety, depression, and, more significantly, cognitive impairments such as learning and memory problems. They can also accelerate aging and cognitive decline in middle-aged individuals, similar to the cognitive issues seen in dementia and Alzheimer's disease in older adults. For these reasons, epilepsy research has begun shifting toward not only suppressing seizures but also developing neuroprotective and disease-modifying therapies. There are many treatments that continue to emerge that aim to slow the progressive neurodegeneration of epilepsy and inhibit seizures.

Recent research on the mechanisms behind molecular and cellular changes suggests that hyperexcitability, which leads to seizures, results from an imbalance between increased excitatory glutamatergic signaling and decreased inhibitory gamma-aminobutyric acid (GABA)-ergic signaling [4]. However, neurodegeneration's role in epilepsy development is becoming more recognized. Key factors driving neurodegeneration in epilepsy include changes in GABAergic neurons and receptors, neuroinflammation, disrupted axonal transport, oxidative stress, excitotoxicity, and other cellular and functional alterations. Using vitamin E, known for its antioxidant, anti-inflammatory, and neuroprotective properties, could be a promising treatment approach for managing epilepsy [5].

Another emerging treatment is targeting microRNAs (miRNAs). miRNAs (miRNAs) are small, noncoding RNAs that help regulate gene expression and play a key role in various biological processes. In epilepsy, changes in the miRNA production pathway have been observed in brain tissue, and experimental seizures can affect miRNAs that control neuronal structure, cell death, inflammation, and ion channels [6]. Targeting specific miRNAs has been shown to influence brain excitability and can either reduce or worsen seizures, suggesting that miRNA-based treatments could be a potential approach for managing epilepsy [7].

Seizure development is increasingly understood as a complex interaction between neurons and glial cells at the tripartite synapse, with neuroinflammation playing a key role in epilepsy. The adenosine triphosphate (ATP)-gated purinergic receptors are found throughout the brain, on both neurons and glial cells. When neuronal activity increases, or during chronic inflammation and cell death, large amounts of ATP are released into the extracellular space, acting as both a neurotransmitter and gliotransmitter. Recent studies suggest that targeting ATP-gated P2 purinergic receptors could effectively influence seizure activity, inflammation, and brain damage caused by seizures [8,9].

Along with the therapeutic treatment options above, this bibliometric analysis examines how the scientific community has responded to the challenge of epilepsy-induced neurodegeneration by tracking the evolution of research trends and their key contributors. It also offers insights into how understanding epilepsy-induced neurodegeneration has grown in recent years and where it is heading.

## Review

Methodology

Bibliometrics is a quantitative method that studies the external features of scientific literature [10]. The Web of Science database was chosen because of its extensive coverage of biomedical research. The preliminary guideline for reporting bibliometric reviews of the biomedical literature (BIBLIO) was used as a framework for constructing this review [11].

The search terms were: Epilepsy (topic) AND treatment (topic) AND neurodegeneration (topic). This combination was used to specifically target treatments for the neurodegeneration seen with epilepsy. No language restriction was applied. All document types related to the search terms were analyzed. No other limitations, such as punctuation and capitalization, were added. The search was limited to the timeframe of January 1, 1989, to November 20, 2025.

Retrieved articles were analyzed for temporal publication trends and for counts pertaining to countries, authors, institutions, and keywords. Visualization maps were produced by exporting the dataset as a tab-delimited file and uploading it into VOSviewer (version 1.6.2, Van Eck & Waltman, 2014). VOSviewer is a software tool for constructing and visualizing bibliometric networks. In maps of bibliographic coupling, co-occurrence of keywords, co-citation, and co-authorship, VOSviewer represents each item as a circle, with its size depicting its significance or contribution. The full counting method was used. The full counting method assigns a weight of one to each link in a network. For example, if a publication has five co-authors, each co-authorship link has a weight of one, and if a keyword appears in 500 documents, that keyword's link strength would be 500. This differs from fractional counting, where a publication's weight is distributed among its co-authors, with each receiving a fractional value. Items are connected by colored links, in which the lengths and thicknesses determine the strength of the connection between them (also known as total link strength).

Results

The search yielded a total of 662 publications. The majority of these publications, 652 (98.5%), were written in English, while others were written in German, Spanish, Russian, and French. One hundred seventy-nine (27%) of these were review articles, and 54% were open access.

Publications took place in 99 countries. The top five countries with publications in this topic include the United States with 675 (32.7%), the People’s Republic of China with 163 (11%), Germany and India with 61 articles each (9.2%), and Italy with 55 (8.3%).

**Figure 1:**
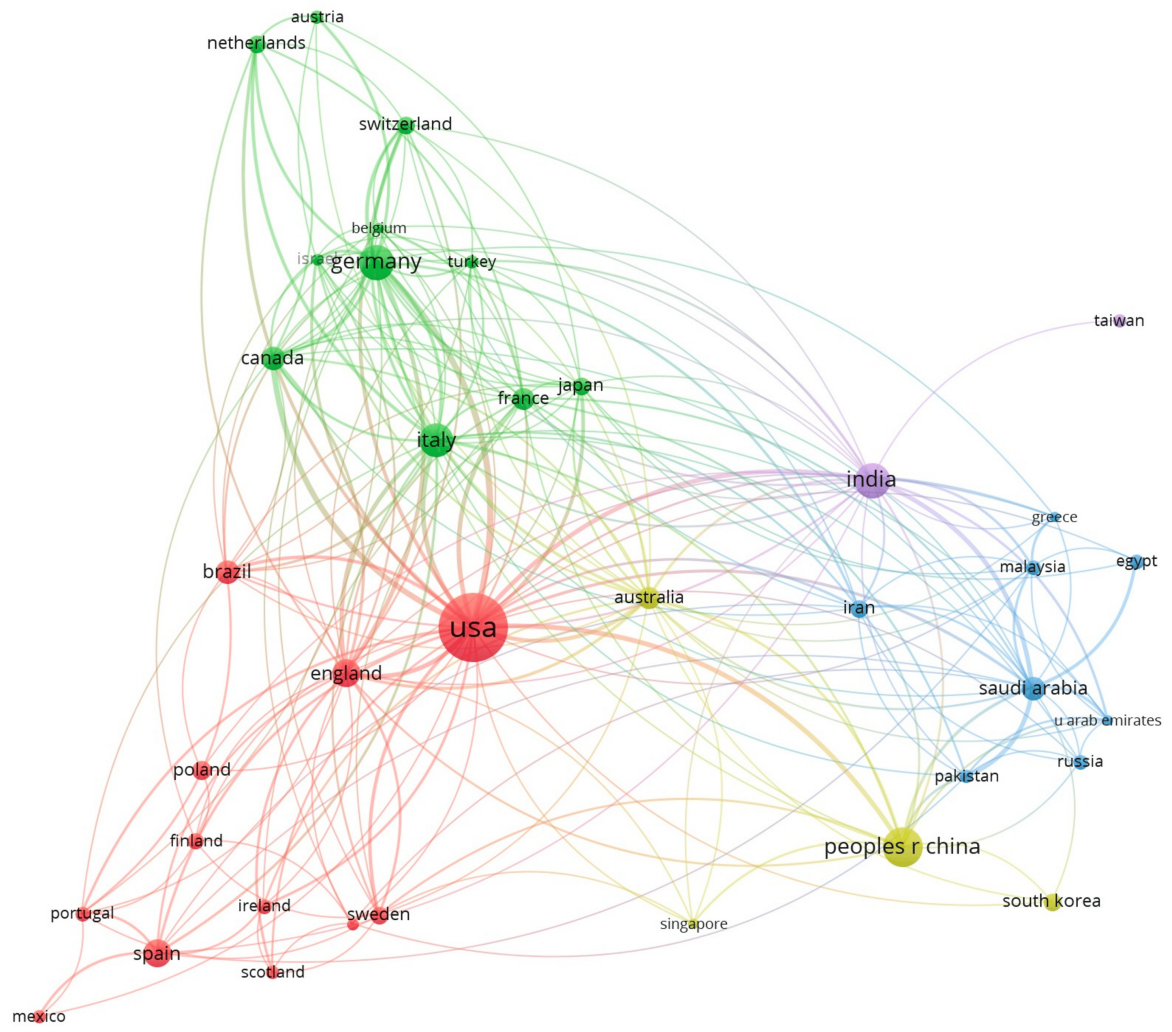
The number of articles written and the international collaboration network among corresponding countries.

The United States also had the strongest international collaboration network with a total link strength of 148, followed by Germany (76), Italy (63), and England (62). The bibliometric map below displays the geographical distribution of publications between the years 1989 and 2025. It shows the number of articles written by each country (based on circle size) and the strength of their international collaboration (Figure 1).

**Figure 2:**
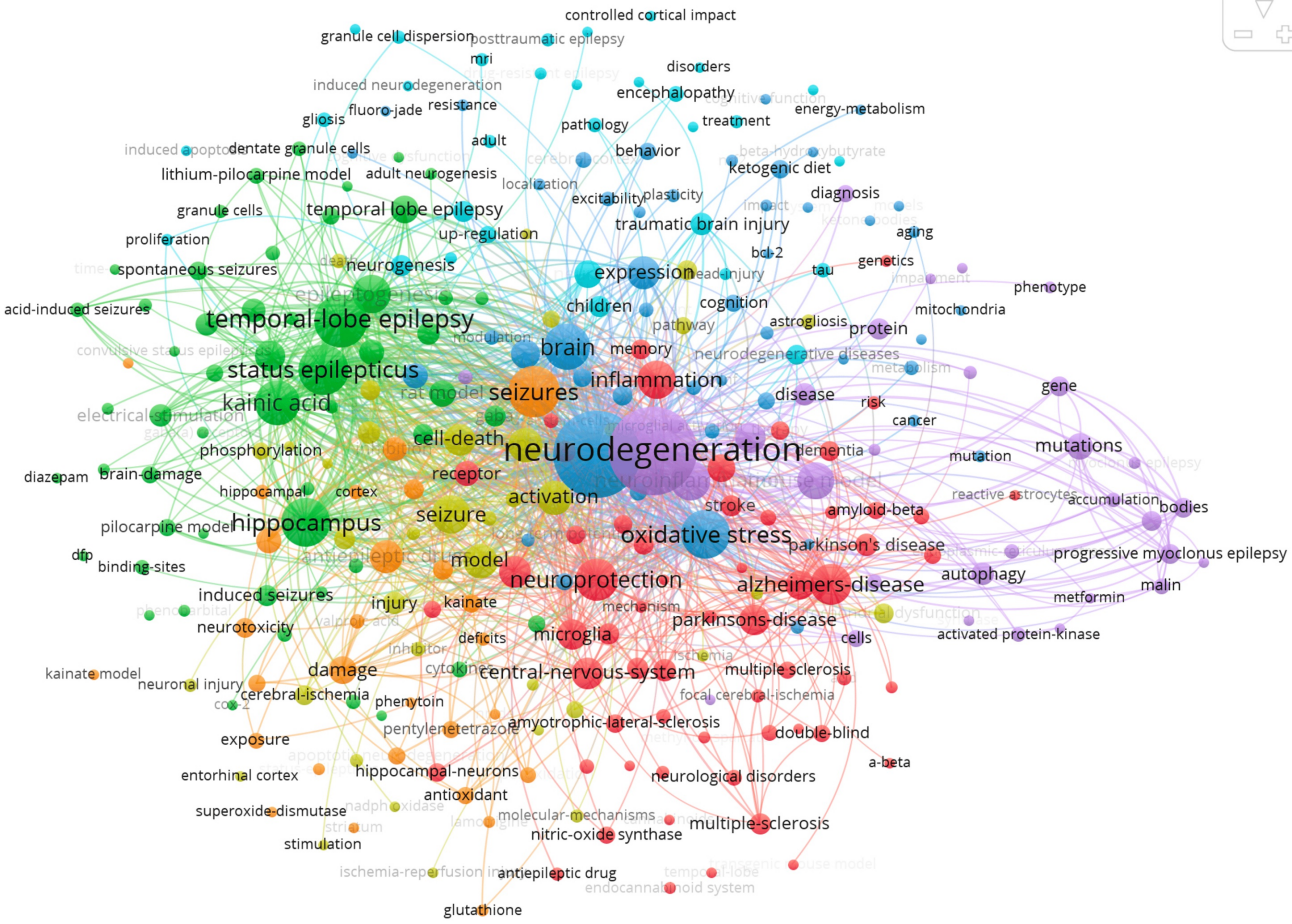
Keyword co-occurrences used in relevant articles published between 1989 and 2025.

Keyword co-occurrence analysis revealed the most frequently used terms in the literature. The core keywords include “epilepsy,” “neurodegeneration,” “seizures,” “hippocampus,” “temporal lobe epilepsy,” “oxidative stress,” and “brain.” These terms formed connected clusters, indicating a high degree of commonality among studies. Also, oxidative stress and neuroinflammation have become increasingly important topics in recent years, suggesting a shift in research priorities toward understanding the molecular and cellular mechanisms of neuronal damage in epilepsy (Figure 2). 

**Figure 3:**
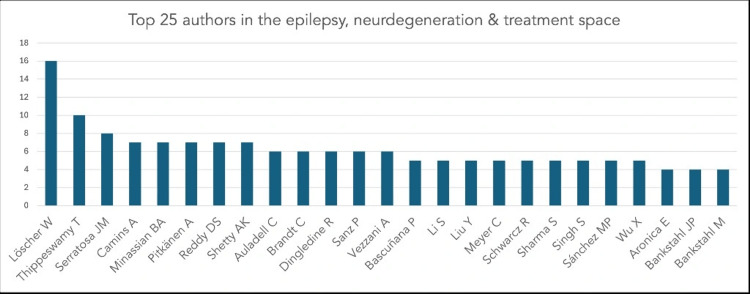
All authors contributing to epilepsy-induced neurodegeneration topics between 1989 and 2025.

Figure 3 depicts the most prolific authors in this field. Highly published contributors such as Wolfgang Löscher, Berge Minassian, and Annamaria Vezzani have been frequently cited for their work on hippocampal damage, seizure mechanisms, and neuroprotective pharmacology (Figure 3). The top five institutions were the University of London, followed by the Institut National de la Santé et de la Recherche Médicale (Inserm), the University of California system, University College London, and the University of Veterinary Medicine Hannover.

**Figure 4:**
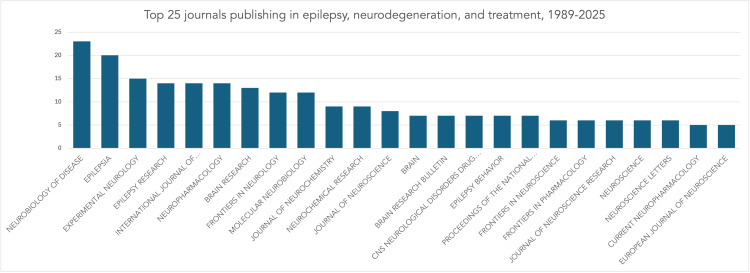
Top 25 journals (by number) with publications in epilepsy and neurodegeneration.

Journal analysis revealed that the majority of the publications appeared in specialized neuroscience and neurology journals such as Epilepsia, Neurobiology of Disease, Neuroscience, International Journal of Molecular Sciences, and Epilepsy Research, which would be expected from the focus of the search (Figure 4). 

Overall, the bibliometric trends illustrate a rapidly expanding field with increasing global interest and international collaboration. The growing emphasis on research of neurodegenerative mechanisms and innovative therapies suggests a promising future for both scientific discovery and clinical application in epilepsy care, as well as the inhibition of neurodegeneration. 

Discussion

This bibliometric analysis provides a comprehensive overview of global research trends in epilepsy-associated neurodegeneration over a 36-year period. The findings demonstrate a substantial expansion of scientific output, reflecting increasing recognition of epilepsy not solely as a disorder of recurrent seizures but also as a condition characterized by progressive neuronal injury, cognitive decline, and network-level dysfunction. The rise in publications since the early 2000s parallels advances in molecular neuroscience, neuroimaging, and biomarker discovery, which have collectively shifted epilepsy research toward mechanisms of neurodegeneration and interventions aimed at neuroprotection.

A key insight from the analysis is the predominance of work originating from the United States, China, and major European research hubs. These findings are consistent with prior bibliometric studies in neurology that have highlighted similar global patterns in epilepsy, Alzheimer’s disease, and neuroinflammation research [12]. The strong international collaboration network centered around the United States likely reflects robust federal research funding, established epilepsy centers, and mature cross-institutional partnerships. Such collaborations are essential for tackling complex topics like neurodegeneration, where multi-omics, large animal models, advanced electrophysiology, and high-resolution imaging benefit from multidisciplinary and multinational expertise.

Our keyword co-occurrence data indicate that earlier research in this domain largely focused on the hippocampus and temporal lobe epilepsy (TLE), paralleling the foundational understanding that hippocampal sclerosis represents one of the most characteristic neuropathological substrates of chronic epilepsy [13]. Over time, however, there has been a notable shift in research priorities toward mechanistic hallmarks of neurodegeneration, including oxidative stress, mitochondrial dysfunction, excitotoxicity, and neuroinflammation. This shift is supported by growing evidence that seizures induce cascades of metabolic stress and inflammatory signaling, leading to neuronal apoptosis, synaptic dysregulation, and altered plasticity [14,15].

The prominence of neuroinflammation-related keywords aligns with accumulating evidence that inflammatory mediators, including cytokines, chemokines, microglial activation pathways, and purinergic receptor signaling, play pivotal roles in epileptogenesis and seizure-induced neurodegeneration. In particular, the purinergic P2X7 receptor has emerged as a promising therapeutic target, with studies suggesting that its blockade may attenuate seizure severity, excitotoxicity, and neuroinflammatory damage [16,17]. The clustering of P2 receptor research identified in this analysis indicates growing interest in glial-neuronal interactions at the tripartite synapse, underscoring a paradigm shift away from neuron-centric models of epilepsy.

The analysis also highlights increasing attention to innovative therapeutic approaches, such as antioxidant therapy and miRNA-based interventions. For example, vitamin E, long studied for its antioxidant and anti-inflammatory properties, has demonstrated neuroprotective potential in experimental epilepsy through reduction of lipid peroxidation and preservation of neuronal architecture [18]. Similarly, miRNAs, which regulate post-transcriptional gene expression, have been shown to modulate pathways involved in axonal integrity, neuroinflammation, cell survival, and ion channel function [19,20]. These findings suggest a future trajectory in which disease-modifying therapies may complement or surpass conventional antiseizure medications, which primarily target neuronal excitability without addressing neuronal loss.

The prominence of experienced authors such as Löscher, Vezzani, and Minassian further underscores the dominant role of established laboratories in shaping the field. Löscher’s work on pharmacoresistance and neuroprotective compounds, Vezzani’s seminal research on neuroinflammation and cytokine biology, and Minassian’s contributions to genetic epilepsies and developmental neurobiology have collectively advanced mechanistic understanding and therapeutic innovation. Their strong citation networks highlight the continued impact of foundational research teams in guiding conceptual and methodological frameworks.

Journal distribution patterns also reflect the field’s interdisciplinarity. Publications span molecular biology, neurology, pharmacology, and genomics journals, illustrating the cross-cutting nature of neurodegeneration research in epilepsy. The substantial presence of articles in Epilepsia, Neurobiology of Disease, and Neuroscience mirrors the clinical-translational continuum from mechanistic discovery to therapeutic evaluation.

Overall, the bibliometric trends support the conclusion that epilepsy-associated neurodegeneration has become a major research frontier with significant clinical implications. As epilepsy is increasingly understood as a progressive network disorder, identifying therapies that prevent or reverse neuronal injury remains a high priority. Continued expansion of global collaboration, integration of high-content molecular technologies, and cross-disciplinary approaches will be critical for developing disease-modifying therapies that go beyond seizure suppression to preserve cognition and long-term neurological health.

## Conclusions

This bibliometric analysis demonstrates substantial and accelerating growth in research focused on epilepsy-related neurodegeneration. Global collaboration networks, established research leaders, and a growing emphasis on molecular mechanisms have shaped the field’s evolution. Increasing attention to neuroinflammation, oxidative stress, miRNA regulation, and neuroprotective therapies reflects a shift toward disease modification rather than seizure control alone. As the understanding of epilepsy expands to include progressive neuronal injury, the development of targeted interventions aimed at preserving brain structure and function becomes critically important. Future multidisciplinary and international collaborations will be essential to advancing therapeutic discovery and improving patient outcomes.
